# Supplementary material for: Machine-learning Prognostic Models from the 2014–16 Ebola Outbreak: Data-harmonization Challenges, Validation Strategies, and mHealth Applications
Source: eClinicalMedicine. 2019 Jun 22;11:54–64. doi: 10.1016/j.eclinm.2019.06.003 (PMC6610774; doi:10.1016/j.eclinm.2019.06.003)
Supplement: Supplementary file 1 — Supplementary material [file mmc1.docx]

**Machine-Learning prognostic models from the 2014-16 Ebola outbreak: data-harmonization challenges, validation strategies, and mHealth applications**

Andres Colubri^1,2,3*^, Mary-Anne Hartley^4,5^, Mathew Siakor^6^, Vanessa Wolfman^6^, August Felix^2^, Tom Sesay^7^, Jeffrey G. Shaffer^8^, Robert F. Garry^9^, Donald S. Grant^10^, Adam C. Levine^6,11*^, Pardis C. Sabeti^1,2,3,12^

^1^ Harvard University, Department of Organismic and Evolutionary Biology. Cambridge, USA

^2^ Broad Institute of MIT and Harvard. Cambridge, USA

^3^ Howard Hughes Medical Institute. Chevy Chase, USA

^4^ University of Lausanne, Faculty of Biology and Medicine. Lausanne, Switzerland

^5^ GOAL Global. Dublin, Ireland

^6^ International Medical Corps. Los Angeles, USA

^7^ Ministry of Health and Sanitation. Freetown, Sierra Leone

^8^ Tulane University, School of Public Health and Tropical Medicine. New Orleans, USA

^9^ Tulane University, Department of Microbiology and Immunology. New Orleans, USA

^10^ Viral Hemorrhagic Fever Program, Kenema Government Hospital. Kenema, Sierra Leone

^11^ Brown University, Warren Alpert School of Medicine. Providence, USA

^12^ Harvard School of Public Health. Boston, USA

^*^ Corresponding authors: [acolubri@fas.harvard.edu](mailto:acolubri@fas.harvard.edu), alevine@internationalmedicalcorps.org

**Keywords:** Ebola Virus Disease, Prognostic Models, Machine Learning, Data Visualization, Severity Score, mHealth, Supportive Care Guidelines, Clinical Intuition

**Abstract**

**Background:** Ebola virus disease (EVD) plagues low-resource and difficult-to-access settings. Machine learning prognostic models and mHealth tools could improve the understanding and use of evidence-based care guidelines in such settings. However, data incompleteness and lack of interoperability limit model generalizability. This study harmonizes diverse datasets from the 2014-16 EVD epidemic and generates several prognostic models incorporated into the novel *Ebola Care Guidelines* app that provides informed access to recommended evidence-based guidelines.

**Methods:** Multivariate logistic regression was applied to investigate survival outcomes in 470 patients admitted to five Ebola treatment units in Liberia and Sierra Leone at various timepoints during 2014-16. We generated a parsimonious model (viral load, age, temperature, bleeding, jaundice, dyspnea, dysphagia, and time-to-presentation) and several fallback models for when these variables are unavailable. All were externally validated against two independent datasets and compared to further models including expert observational wellness assessments. Models were incorporated into an app highlighting the signs/symptoms with the largest contribution to prognosis.

**Findings:** The parsimonious model approached the predictive power of observational assessments by experienced clinicians (Area-Under-the-Curve, AUC=0⋅70-0⋅79, accuracy=0⋅64-0⋅74) and maintained its performance across subcohorts with different healthcare seeking behaviors. Age and viral load contributed >5-fold the weighting of other features and including them in a minimal model had a similar AUC, albeit at the cost of specificity.

**Interpretation:** Clinically guided prognostic models can recapitulate clinical expertise and be useful when such expertise is unavailable. Incorporating these models into mHealth tools may facilitate their interpretation and provide informed access to comprehensive clinical guidelines.

**Funding:** Howard Hughes Medical Institute, US National Institutes of Health, Bill & Melinda Gates Foundation, International Medical Corps, UK Department for International Development, and GOAL Global

**Research in context**

**Evidence before this study**. The recent Ebola virus disease (EVD) outbreaks have highlighted the need for field-deployable patient management tools adaptable to its heterogeneous spectrum of pathology across widely varying environments and resources. In 2015, a WHO-led assessment of shortcomings in the initial response to the unfolding epidemic noted that “better information was needed to understand best practices in clinical management” and “innovations in data collection should be introduced, including mHealth communications”. Despite the availability of numerous clinical guidelines, their digital accessibility is poorly adapted to field conditions and difficult to navigate or read on mobile phones. Further, information is distributed across various guidelines, quickly outdated, and undirected reading is often overly detailed or too generic for the patient at hand. On the Google Play store, the search term “Ebola” returned just three EVD-specific applications directed at clinicians, only one of which assimilated guidelines from 2014 within their current structure. Machine learning prognostic models could improve understanding and personalized use of evidence-based guidelines by helping to prioritize recommended interventions according to the prognostic importance of each clinical feature present in the individual patient. We searched PubMed without date limits for various permutations of terms representing “prognostic”, “machine learning”, “mHealth”, “guidelines” and “Ebola”. Less than ten EVD symptomatic prognostic models were found, with most constructed on single-site cohorts without external validation. While one provided a paper scorecard, none were integrated in an mHealth tool or made use of machine learning.

**Added value of this study**. This study is based on the largest and most diverse clinical EVD dataset available to date, comprising 470 confirmed EVD cases from five different locations in Sierra Leone and Liberia, and a further 264 cases from two independent datasets for external validation. It demonstrates how interoperability between diverse datasets can be achieved through data harmonization approaches and constructs a family of flexible externally validated prognostic models that are able to approximate observational wellness assessments made by experienced clinicians. We further integrate these models in the first field-deployable mobile app for EVD prognostication, which enables informed access to recommended guidelines.

**Implications of all the available evidence**

The *Ebola Care Guidelines* app benchmarks an approach to generating evidence-based mobile clinical management tools to improve training and informed adherence to protocol, where a personalized predictive support system organizes clinical procedures more effectively around patient data.

**Introduction**

The 2014-2016 outbreak of EVD caused a worldwide health crisis with more than 28,000 cases and 11,000 deaths, the vast majority of which occurred in the West African countries of Liberia, Sierra Leone, and Guinea. The recent ongoing outbreak in the Democratic Republic of the Congo (1) is evidence of the threat posed by EVD, even with the availability of experimental vaccines. Of particular concern, is the presence of outbreaks in regions with limited medical coverage such as the active conflict zone affected by the current outbreak (2). Despite its notoriety as a deadly disease, the pathology of EVD includes a range of outcomes, spanning from asymptomatic infection to complex organ failure. On the few cases treated in high-income countries, case fatality ratios (CFRs) of under 20% were achievable, revealing the importance of resources in determining prognosis (3). Prioritizing time and material resources for high-risk patients in remote and low-resource settings is one approach to decrease overall mortality when subject to such constraints (4). A complementary approach is to use tools providing clinical instructions for management, training, and improved protocol adherence (5, 6).

We previously introduced the use of prognostic models that can be deployed as mobile apps for the purpose of risk stratification in EVD (7). Our original models were developed on the single publicly available dataset at that time by Schieffelin et al. (8), which includes 106 Ebola-positive patients admitted at Kenema Government Hospital (KGH). These models outperformed simpler risk scores and allowed users to choose from various sets of predictors depending on the available clinical data. While this study showed the potential for such an approach, the models were limited by the narrow geographical and temporal scope of the relatively small and incomplete dataset. Further, the prototype app in which the models were packaged displayed only the severity score of the patient without further guidance. We thus sought to create models with greatly expanded geographic relevance packaged in a new app that could provide risk-based guidance to health workers particularly in limited-resource settings. The models are derived from the largest and most diverse EVD dataset published to date, comprised of 470 confirmed cases from five treatment centers spread across Sierra Leone and Liberia at various timepoints during the 2014-16 epidemic (provided by the international Medical Corps, IMC) and further externally validated on two datasets of 264 cases (comprising the 106 KGH dataset and an additional 158 provided by GOAL global).

Despite the availability of numerous clinical guidelines for EVD, their digital accessibility is poorly adapted to field conditions where their book-like formatting makes for awkward navigation and reading on mobile devices. Further, the static nature of these documents makes them quickly outdated, especially in the rapidly evolving context of an epidemic where new recommendations are often fragmented across research papers and field reports that are challenging and sometimes impossible for health workers to access. Our goal is not only to provide an updatable platform on which care guidelines could be centralized, but also to personalize the prioritization of recommendations based on the severity score of the individual patient, as predicted by validated prognostic models. Such tailored guidance can be achieved by objectively highlighting the symptom-based interventions that are most relevant given all the available information about the patient during triage.

**Methods**

**IMC Patient Cohort and Data Collection**

Prognostic models were based on data collected from 470 patients at five ETUs operated by IMC in Liberia (n=178, 38%) and Sierra Leone (n=292, 62%) between September 15, 2014 and September 15, 2015. The ETUs were located at Lunsar (Port Loko District), Kambia (Kambia District), and Makeni (Bombali District) in Sierra Leone, and at Suakoko (Bong County) and Kakata (Margibi County) in Liberia. The majority of the patients presented directly to these centers without secondary referrals from holding units. The overall Case Fatality Ratio (CFR) across the 5 ETUs was 58%.

Trained health workers recorded patient demographic, clinical, and support data at least daily from admission to discharge on standardized paper forms. Collection and archival protocols are detailed in Roshania et al (9). The clinical and lab protocols were mostly consistent across the five ETUs, making it possible to aggregate individuals into a single cohort. Notable exceptions were wellness scale (WS) and body temperature, both of which were only recorded in Sierra Leonean ETUs. WS is an observational assessment assigned by experienced clinicians on a scale ranging from 0 (cured) to 5 (very sick) as seen in Table 1. WS was recorded for 223 of the 292 patients treated at the three ETUs in Sierra Leone and was imputed for the 89 Sierra Leonean patients without it. The categorical variable fever, available for all ETUs, was used to guide imputation of body temperatures.

The cycle threshold (CT) value is an inversely proportional proxy of viral load, with a cut-off of 40 cycles considered as negative. These values were calculated from PCRs performed on admission or from the second PCR when the first was missing, performed no later than two days after admission and affecting a total of 28 patients. The mean CT value of these patients (25) was not significantly different from the rest of the cohort (24) in crude analyses or when stratified by outcome. PCR data was normalized across sites to adjust for analytical bias between laboratories (described below).

**Table 1. Wellness scale.** Interpretation of the 0-to-5 observational scale of patient wellness at the Sierra Leonean ETUs.

| **Wellness Scale** | **Interpretation** |
| --- | --- |
| **0** | Cured |
| **1** | Well: no symptoms: drinks and eats okay |
| **2** | Few symptoms: drinks and eats okay |
| **3** | Moderate symptoms: can walk, sit, and feed independently |
| **4** | Sick: needs help to be fed, drink, and take medications |
| **5** | Very sick: needs IV fluids and medications, lots of assistance |

**External Validation cohorts**

External validation was performed on two independently collected datasets from Sierra Leone. The KGH dataset described by Schieffelin et al. (8) is the only such database to be made publicly available at the time of this study (https://dataverse.harvard.edu/dataverse/ebola). It includes 106 EVD-positive cases treated at KGH between 25 May and 18 June 2014, where CFR was 73%. Signs and symptoms at triage were available for 44 patients and viral load was determined in 58 cases. Missingness across the dataset was 78%. The GOAL dataset described by Hartley et al. (10, 11) includes 158 EVD-positive cases treated at the GOAL-Mathaska ETU in Port Loko between December 2014 and June 2015, where the CFR was 60%. RT-PCR results and detailed sign and symptom data were available for all 158 patients. Missingness across the dataset was 1%.

**Acquisition, Transformation and Normalization of Cycle Threshold Values**

Further details on the assays and processing methodologies are provided in the supplementary materials (PCR Lab Notes section in suppl. mat). RT-PCR data were obtained from four laboratories. Liberian ETUs were served by the United States Naval Medical Research Center (NMRC) Mobile Laboratory in Bong County. In Sierra Leone, the Lunsar, Makeni and GOAL ETU’s were served by Public Health England (PHE), while a Nigerian Lab handled samples from the Kambia ETU. KGH had on-site laboratory support. Inconsistent with other sites, the KGH PCR data was reported as viral load (VL, in copies/ml) only. To harmonize measures, VL was transformed to CT according to the standard qPCR curve log(VL) = m×CT + c0, such that the minimum VL in the KGH dataset corresponded to the maximum CT in the aggregated IMC dataset, and vice versa. The accuracy of the transformation was supported by the observations that its limit of detection aligned to the IMC dataset, (12-14) and also that the curve corresponded to its expected form, where a ≈10-fold increase in Ebola VL corresponded to a 3-point decrease in CT (15). Based on this relationship, -3/m in our formula should be close to 1, which is indeed the case (-3/m=0⋅976 using the m and c0 constants derived from the KGH and IMC data). Transformation results were not dependent on the geographical origin of the IMC data.

Geographical origin did, however, have a significant (P<0⋅0001) impact on CT distribution across sites (Suppl. Figure S1), where IMC ETU’s had a mean of 21⋅82±5⋅16 in Sierra Leone and 27⋅67±5⋅45 in Liberia. In the KGH and GOAL external validation cohorts, these values were 26⋅05±6⋅00 and 22⋅20±4⋅31, respectively. A possible reason for this discrepancy is the differing methods of RT-PCR in each site (TaqMan in the Liberian ETUs, commercial Altona and in-house “Trombley” in the Sierra Leonean ETUs). To correct for such analytical bias, and ensure the results were internally consistent at each site and thus comparable between sites, we normalized the CT values from each site/group of sites by feature-scaling (subtracting the mean and dividing by the standard deviation). As the differences in CT could also arise from differences in care-seeking behaviors, the normalization of CT values within sites would also work towards reducing the effect of this potential confounder.

**Ethical approval**

The Sierra Leone Ethics and Scientific Review Committee, the University of Liberia – Pacific Institute for Research & Evaluation Institutional Review Board, the Lifespan (Rhode Island Hospital) Institutional Review Board, and the Harvard Committee on the Use of Human Subjects provided ethical approval for this study and exemption from informed consent. A data sharing agreement was approved by IMC and the Broad Institute, following IMC’s Research Review Committee Guidelines (https://internationalmedicalcorps.org/document.doc?id=800).

**Exploratory and Bivariate Analysis**

Analyses were undertaken in R version 3⋅5⋅1. The primary outcome was final disposition (death or survival). The outcome of five patients was missing due to being transferred to another facility. We carried out an initial bivariate analysis of all factors against outcome, using the χ^2^ test with Yates correction for the binary variables, and the point biserial correlation test for numerical variables.

**Multiple imputation**

Across all the potential predictors in the dataset, 22% of fields had missing values. Predictor exclusion was limited to those having more than 50% missingness so as to minimize bias in the regression coefficients (16). The remaining missing values were assessed for their suitability for imputation, using Little’s MCAR test (R package BaylorEdPsych), which tests for bias in the missing values or whether they are “missing completely at random” (MCAR) (17). Multiple imputation was undertaken with the aregImpute function from the R package Hmisc (18), which generates a Bayesian predictive distribution from the known data, and outputs a number N of imputed datasets. Each missing value in the i^th^ imputation is predicted from an additive model fitted on a bootstrap sample with replacement from the original data. We set N=100, well above standard imputation guidelines (19).

**Machine learning Feature Selection and Multivariate modelling**

Several logistic regression models were constructed to predict the binary outcome death/survival from the demographic, clinical (signs and symptoms), and laboratory data (viral load and malaria rapid test results) collected at triage. The predictors for the models were selected in a machine learning variable-selection process, where an initial set of candidate factors associated with death in EVD were submitted to penalized logistic regression using the R package Glmnet (20) with Elastic Net regularization. To define a non-redundant, parsimonious subset of predictors, variables with a coefficient >0 in at least half of the penalized models were retained. As marginal associations can become less significant when accounting for confounding dependencies in such multivariate models, the process tended to include variables with weak correlations to the outcome in bivariate analysis, such as bleeding and dyspnea, and elimination of variables that had a low P-value, like conjunctivitis. Using these variables, we constructed a family of non-penalized logistic regression models using the lrm function from the R package rms (21). This included a parsimonious model (using all the variables obtained from the selection process), and several fallback models that can be applied on smaller subsets of demographic information, clinical features and laboratory results, for use when more limited data is available at triage. This approach has been shown to outperform predictive value imputation, which consists of having only one full model and imputing missing values at prediction time using the data distribution from the training set (22). Each final model in the family was obtained by fitting N copies of the model on each imputed dataset, and then averaging those copies into a single model using the fit.mult.impute function in Hmisc. Internal validation was performed using bootstrap resampling and model performance is presented by the area under the curve (AUC), McFadden’s pseudo-R^2^, Brier score, accuracy, sensitivity and specificity. A prediction was classified as death when the score from the model was over the 0⋅5 threshold. Confidence intervals (CI) of all the performance estimates were calculated using Fisher’s transformation (23). Odds ratios (OR) from logistic regression were converted to risk ratios (RRs) according to Zhang and Yu (24).

**Results**

**Prognostic Potential and Prevalence of Signs and Symptoms Recorded at Triage**

Triage symptoms reported by over 50% of fatal Ebola patients were anorexia/loss of appetite, fever, asthenia/weakness, musculoskeletal pain, headache and diarrhea (Table 2A). Prevalence of several triage symptoms was notably different between fatal and non-fatal outcomes, as can be seen by comparing their ranking (Suppl. Figure 2A) or their differential prevalence (Suppl. Figure 2B). Few variables were significantly associated with patient outcome. Only CT, age (Table 2B), and jaundice (Table 2A) were associated with death at a level of P<0⋅05, while conjunctivitis, confusion, dyspnea, headache, and bleeding were weakly associated at P<0⋅15.

**Table 2. Bivariate analysis.** Correlation between binary (A) and continuous (B) clinical variables and the outcome of death. For binary variables, crude marginal risk-ratios (RR) were obtained from the 2x2 contingency table. For continuous variables, the odds ratios (OR) correspond to inter-quartile range changes in the variables when used as the only predictor of death (and 10-years increase in the case of age).

*#: variables not recorded at the Sierra Leonean ETUs, §: variables not recorded at the Liberian ETUs.*

**A**

| **Variable** | **Total (%)** | **Non-fatal (%)** | **Fatal (%)** | **Missing (%)** | **RR**  **(95% CI)** | **P-value** |
| --- | --- | --- | --- | --- | --- | --- |
| **Jaundice** | **24/464 (5)** | 4/197 (2) | 20/267 (7) | 1/470 (0) | 1⋅06  (1⋅02, 1⋅10) | 0⋅016 |
| **Conjunctivitis** | **128/464 (27)** | 64/197 (32) | 64/267 (23) | 1/470 (0) | 0⋅89  (0⋅79, 1⋅00) | 0⋅054 |
| **Coma#** | **5/178 (2)** | 0/83 (0) | 5/95 (5) | 292/470 (62) | 1⋅05  (1⋅00, 1⋅11) | 0⋅096 |
| **Confusion#** | **16/178 (8)** | 4/83 (4) | 12/95 (12) | 292/470 (62) | 1⋅09  (1⋅00, 1⋅19) | 0⋅120 |
| **Dyspnea** | **109/464 (23)** | 39/197 (19) | 70/267 (26) | 1/470 (0) | 1⋅09  (0⋅98, 1⋅20) | 0⋅133 |
| **Headache** | **268/464 (57)** | 122/197 (61) | 146/267 (54) | 1/470 (0) | 0⋅84  (0⋅67, 1⋅05) | 0⋅142 |
| **Bleeding** | **26/464 (5)** | 7/197 (3) | 19/267 (7) | 1/470 (0) | 1⋅04  (0⋅99, 1⋅08) | 0⋅148 |
| **Asthenia/Weakness** | **334/464 (71)** | 135/197 (68) | 199/267 (74) | 1/470 (0) | 1⋅24  (0⋅92, 1⋅65) | 0⋅187 |
| **Diarrhea** | **234/430 (54)** | 96/187 (51) | 138/243 (56) | 35/470 (7) | 1⋅13  (0⋅92, 1⋅38) | 0⋅304 |
| **Malaria§** | **49/225 (21)** | 17/94 (18) | 32/131 (24) | 241/470 (51) | 1⋅08  (0⋅95, 1⋅24) | 0⋅331 |
| **Dysphagia** | **112/464 (24)** | 43/197 (21) | 69/267 (25) | 1/470 (0) | 1⋅05  (0⋅95, 1⋅17) | 0⋅374 |
| **Vomiting** | **197/464 (42)** | 87/197 (44) | 110/267 (41) | 1/470 (0) | 0⋅95  (0⋅81, 1⋅11) | 0⋅587 |
| **Nausea§** | **94/286 (32)** | 35/114 (30) | 59/172 (34) | 179/470 (38) | 1⋅05  (0⋅90, 1⋅24) | 0⋅613 |
| **Abdominal Pain** | **203/464 (43)** | 89/197 (45) | 114/267 (42) | 1/470 (0) | 0⋅96  (0⋅81, 1⋅13) | 0⋅662 |
| **Bone/Muscle/Joint Pain** | **272/465 (58)** | 118/197 (59) | 154/268 (57) | 0/470 (0) | 0⋅94  (0⋅76, 1⋅17) | 0⋅666 |
| **Throat Pain#** | **55/178 (30)** | 24/83 (28) | 31/95 (32) | 292/470 (62) | 1⋅06  (0⋅87, 1⋅28) | 0⋅709 |
| **Cough#** | **61/178 (34)** | 30/83 (36) | 31/95 (32) | 292/470 (62) | 0⋅95  (0⋅77, 1⋅17) | 0⋅738 |
| **Hiccups** | **55/464 (11)** | 22/197 (11) | 33/267 (12) | 1/470 (0) | 1⋅01  (0⋅95, 1⋅08) | 0⋅805 |
| **Rash#** | **8/178 (4)** | 3/83 (3) | 5/95 (5) | 292/470 (62) | 1⋅02  (0⋅96, 1⋅08) | 0⋅867 |
| **Chest Pain#** | **88/178 (49)** | 41/83 (49) | 47/95 (49) | 292/470 (62) | 1  (0⋅75, 1⋅34) | 0⋅889 |
| **Photophobia#** | **24/178 (13)** | 11/83 (13) | 13/95 (13) | 292/470 (62) | 1  (0⋅89, 1⋅13) | 0⋅892 |
| **Anorexia/Poor Appetite** | **316/464 (68)** | 135/197 (68) | 181/267 (67) | 1/470 (0) | 0⋅98  (0⋅75, 1⋅28) | 0⋅946 |
| **Fever** | **349/464 (75)** | 148/197 (75) | 201/267 (75) | 1/470 (0) | 1⋅01  (0⋅73, 1⋅39) | 0⋅944 |

**B**

| **Variable** | **Non-fatal**  **(mean, 95% CI)** | **Fatal**  **(mean, 95% CI)** | **Missing**  **fraction (%)** | **Pearson's R** | **OR**  **(95% CI)** | **P-value** |
| --- | --- | --- | --- | --- | --- | --- |
| **Cycle Threshold** | 26⋅72  (15⋅92, 37⋅52) | 22⋅23  (11⋅18, 33⋅28) | 137/470 (29) | -0⋅37 | 0⋅33  (0⋅23, 0⋅47) | <0⋅0001 |
| **Wellness Scale§** | 2⋅49  (0⋅82, 4⋅17) | 3⋅20  (1⋅17, 5⋅22) | 247/470 (52) | 0⋅34 | 4⋅57  (2⋅44, 8⋅58) | <0⋅0001 |
| **Patient Age** | 28⋅49  (0⋅00, 58⋅72) | 32⋅03  (0⋅00, 72⋅10) | 4/470 (1) | 0⋅09 | 1⋅33  (1⋅01, 1⋅74) | 0⋅043 |
| **Body Temperature§** | 37⋅41  (35⋅50, 39⋅32) | 37⋅67  (35⋅34, 40⋅01) | 265/470 (56) | 0⋅12 | 1⋅39  (0⋅94, 2⋅06) | 0⋅099 |
| **Time-to-presentation** | 4⋅28  (0⋅00, 12⋅03) | 4⋅20  (0⋅00, 9⋅90) | 108/470 (22) | -0⋅01 | 0⋅97  (0⋅76, 1⋅25) | 0⋅83 |

**Multivariate Logistic Regression Models**

**Variable selection.** Applying a machine learning selection model described in the methods, we obtained a set of variables including patient age, Ebola-specific PCR result (CT), and several clinical features recorded at triage (body temperature, bleeding, jaundice, dyspnea, dysphagia, and time-to-presentation (TTP)).

**Multiple imputation.** Before procedure, selected variables were assessed for bias. We applied restricted cubic splines to model the non-linear relationships between CFR and age (Suppl. Figure S2A) and CFR and body temperature (Suppl. Figure S2B). The actual values of age and temperature were the input of the spline fitting procedure, as implemented in the Hmisc package. The lack of a saddle in the CFR vs CT plot (Suppl. Figure S3) suggested that cubic splines were not required to model CT. Following Hartley’s (10) observation that viral load can act as a confounding factor for outcome and TTP (where patterns in early viral load directs care-seeking behavior as well as determines outcome), we added a CT x TTP interaction. While non-random patterns of missing values were found when considering all data, Little’s MCAR test was satisfied at P=0⋅05 when considering the Sierra Leonean and Liberian records separately, thus identifying geographical location as the main source of non-randomness. The remaining weak non-random patterns for missing fields in the TTP variable in Sierra Leonean records (P=0⋅03) could be explained by low CT values. Inclusion of CT in the model controlled for this effect and ensured randomness within each subset of CT (high versus low).

**Model performance.** In addition to the parsimonious model (using all features described above), we constructed three fallback models for use when certain features were unavailable. The first “parsimonious without temperature” model was identical to the parsimonious but with backwards elimination of body temperature. A second “clinical-only” model selected only clinical features after omitting CT from the initial list of variables before the machine learning selection step. This model included jaundice, bleeding, dyspnea, dysphagia, asthenia/weakness, and diarrhea. A final “minimal” model only incorporated CT and age, which were the strongest individual predictors of outcome, as observed in our data and reported by other researchers (25). Table 3 contains the validation indices and their 95% CIs. Most indices are very similar between the parsimonious and minimal models, with largely overlapping CIs. The specificity and R^2^ are higher in the parsimonious models, while the sensitivity is slightly higher in the minimal model. The addition of temperature consistently increases performance across all indices, but only by a minor amount. The clinical-only model shows consistently lower performance. Detailed description of all the models are provided in Suppl. Table S1.

The calibration curves comparing the predicted and actual probabilities of death (Figure 1) suggest that the two parsimonious models represent the actual probabilities well, with some underestimation for low risk patients. The minimal and clinical-only models are less calibrated, with the former underestimating the actual probabilities at both the low- and high-risk ends, and the later overestimating the actual probabilities for a wide range of risks above 60%.

**Table 3. Validation indices for the prognostic models.** These indices include AUC, McFadden’s pseudo R^2^ goodness-of-fit index, Brier score, overall accuracy, sensitivity, and specificity. The means and 95% confidence intervals were obtained with 200 iterations of bootstrap resampling.

|  | **Parsimonious**  **(95% CI)** | **Parsimonious**  **w/out temp.**  **(95% CI)** | **Clinical-only**  **(95% CI)** | **Minimal**  **(95% CI)** |
| --- | --- | --- | --- | --- |
| **AUC** | 0⋅75  (0⋅70, 0⋅79) | 0⋅74  (0⋅69, 0⋅78) | 0⋅64  (0⋅58, 0⋅69) | 0⋅76  (0⋅71, 0⋅80) |
| **R^2^** | 0⋅22  (0⋅17, 0⋅27) | 0⋅21  (0⋅16, 0⋅25) | 0⋅12  (0⋅09, 0⋅15) | 0⋅16  (0⋅13, 0⋅21) |
| **Brier** | 0⋅21  (0⋅16, 0⋅25) | 0⋅21  (0⋅16, 0⋅26) | 0⋅24  (0⋅19, 0⋅29) | 0⋅21  (0⋅16, 0⋅25) |
| **Accuracy** | 0⋅69  (0⋅64, 0⋅74) | 0⋅68  (0⋅63, 0⋅73) | 0⋅60  (0⋅54, 0⋅66) | 0⋅68  (0⋅63, 0⋅73) |
| **Sensitivity** | 0⋅80  (0⋅75, 0⋅84) | 0⋅79  (0⋅74, 0⋅83) | 0⋅70  (0⋅64, 0⋅75) | 0⋅81  (0⋅76, 0⋅85) |
| **Specificity** | 0⋅57  (0⋅51, 0⋅63) | 0⋅55  (0⋅49, 0⋅61) | 0⋅51  (0⋅44, 0⋅57) | 0⋅52  (0⋅46, 0⋅58) |


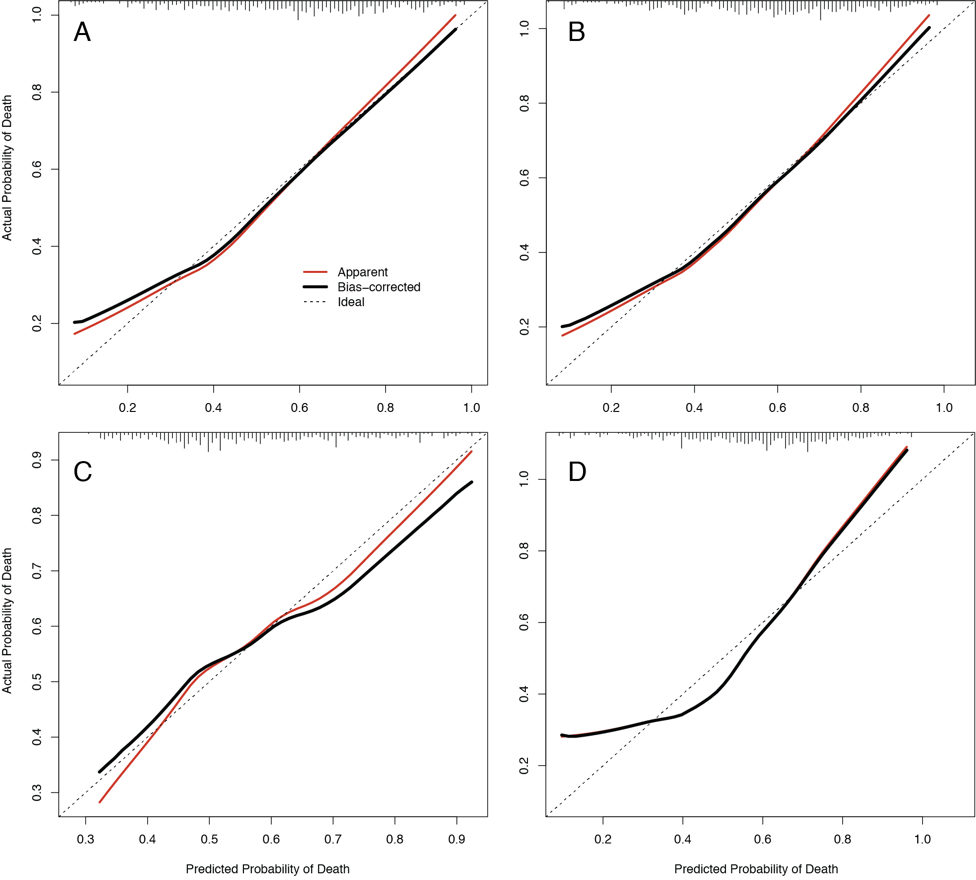


**Figure 1. Bootstrap overfitting-corrected calibration curve**. Estimated for the four prognostic models: parsimonious (A), parsimonious without body temperature (B), clinical-only (C), and minimal (D). Each plot contains the rug chart at the top showing the distribution of predicted risks. The dotted line represents the apparent calibration curve and the solid line shows the optimism-corrected calibration. A perfectly-calibrated model will fall along the diagonal. Generated with the calibrate function in the rms package.

The ranking of all of the variables by their importance in the parsimonious model, as measured by the Wald χ^2^ statistic, reveals that the most important variables are CT and patient age, with jaundice and bleeding coming in at a distant third and fourth place respectively, followed by body temperature and the CT-TTP interaction (Figure 2A). The odds ratios (Figure 2B) indicate that presentation of either jaundice or bleeding are associated with more than a doubling of the odds of death, although their prevalence is low at 5% (Table 2). Transformation of the odds risks into risk ratios (Suppl. Table S1) yields a more reasonable estimate of 4% increase in risk associated to the occurrence of those symptoms at presentation.

**Temporal flexibility of models.** To test the temporal flexibility of models we compared the predictions from the parsimonious model when using the CT value from day 0 versus day 2, for those patients for whom these CT values where available. There were 84 patients in the d0 subcohort (CFR: 63%) and 14 patients in the d2 subcohort (CFR: 64%). The model performed similarly on these cohorts (Suppl. Table S2A) with 15 misclassifications using d0 CT reads subcohort, and 3 using d2 CT reads. Further, comparing patients with slow (>3d) or rapid (≤3d) times to presentation, we see little difference (Suppl. Table S2B) in performance parameters for the parsimonious model.

**
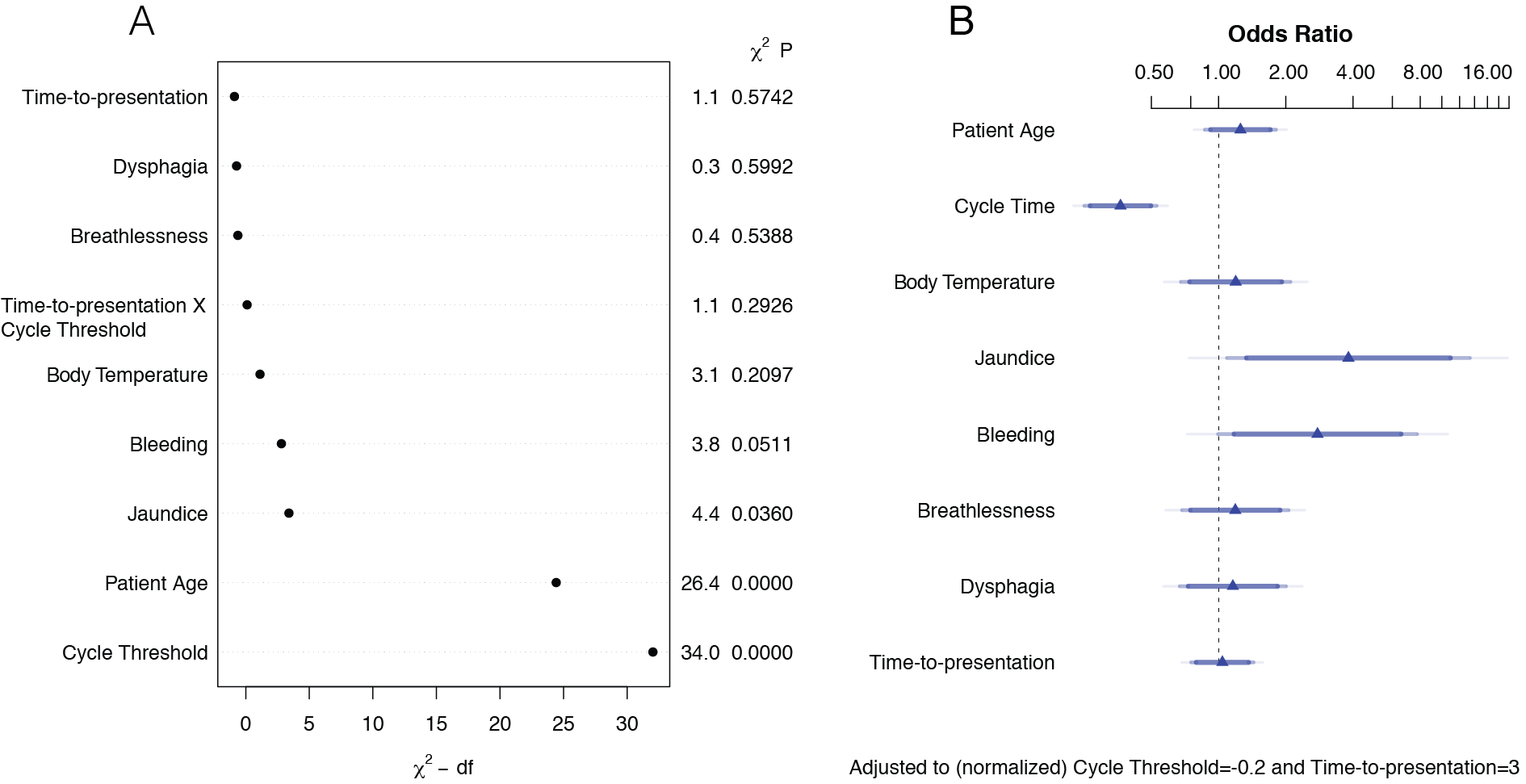
**

**Figure 2. Evaluation of predictor variables in the parsimonious model.** Ranking of the variables according to their predictive importance in the model, as measured by the χ^2^-d.f. (degrees of freedom) statistic (A). Odds ratios for all the variables, using interquartile-range odds ratios for continuous features, and simple odds ratios for categorical features (B). Generated with the anova.rms and summary functions in the rms and base packages in R.

**External validation**

External validation on the 158 EVD-positive patients in the GOAL dataset shows that the four models described earlier improve their performance with respect to the internal validation. CT was missing in 37 patients, and both CT and TTP were missing in 53, so the parsimonious models were validated on 105 patients and the minimal model on 121. The clinical-only model was validated on all the 158 patients. We obtained AUCs of 0⋅82, 0⋅84, 0⋅73, and 0⋅82 for the parsimonious, parsimonious without temperature, clinical-only, and minimal (Table 4A). In terms of the accuracy, sensitivity, and specificity, all the models, with the exception of the clinical-only, performed similarly well with accuracies around 74%. Excluding again the clinical-only model, sensitivity ranged between 78% and 82%, while specificity was lowest for the minimal model at 61% and highest for the parsimonious model at 68%. Therefore, it is not possible to pick an overall best model between the three including CT but depending on what is the priority in the predictive task, higher sensitivity or specificity, one could favor either the parsimonious or the minimal.

For the KGH dataset (Table 4B), only 11 patients had enough complete records to validate the parsimonious models, but after multiple imputation, all the 106 KGH cases could be used. As it was observed in the first validation, all models with the exception of the clinical-only, exhibited consistent performance in terms of AUC and overall accuracy, with analogous differences in terms of specificity and sensitivity. The parsimonious models have higher specificity, while the minimal is more sensitive. The impact of imputation was inconsequential.

**Table 4. External validation on the GOAL (A) and KGH (B) datasets.** The AUC, Brier, accuracy, sensitivity, specificity, positive predictive values (PPV) and negative predictive values (NPV) indices were calculated (A) on all the records from the GOAL dataset with complete data and (B) all the records from the KGH dataset that had enough data to evaluate the clinical-only and minimal models. The results for the parsimonious models were obtained after imputing missing values in the KGH patients, and the performance indices include the mean and standard deviation over 100 multiple imputations.

**A**

|  | **Parsimonious** | **Parsimonious**  **w/out temp.** | **Clinical-only** | **Minimal** |
| --- | --- | --- | --- | --- |
| **AUC** | 0⋅82 | 0⋅84 | 0⋅73 | 0⋅82 |
| **Brier** | 0⋅17 | 0⋅16 | 0⋅21 | 0⋅17 |
| **Accuracy** | 0⋅74 | 0⋅75 | 0⋅66 | 0⋅74 |
| **Sensitivity** | 0⋅78 | 0⋅82 | 0⋅77 | 0⋅82 |
| **Specificity** | 0⋅68 | 0⋅63 | 0⋅52 | 0⋅61 |
| **PPV** | 0.81 | 0⋅80 | 0⋅66 | 0⋅79 |
| **NPV** | 0.63 | 0⋅67 | 0⋅65 | 0⋅66 |

**B**

|  | **Parsimonious**  **(SD)** | **Parsimonious**  **w/out temp. (SD)** | **Clinical-only** | **Minimal** |
| --- | --- | --- | --- | --- |
| **AUC** | 0⋅78 (0⋅04) | 0⋅78 (0⋅04) | 0⋅72 | 0⋅82 |
| **Brier** | 0⋅19 (0⋅01) | 0⋅18 (0⋅01) | 0⋅22 | 0⋅17 |
| **Accuracy** | 0⋅73 (0⋅03) | 0⋅72 (0⋅03) | 0⋅59 | 0⋅74 |
| **Sensitivity** | 0⋅73 (0⋅03) | 0⋅73 (0⋅03) | 0⋅56 | 0⋅82 |
| **Specificity** | 0⋅88 (0⋅03) | 0⋅86 (0⋅02) | 0⋅75 | 0⋅61 |
| **PPV** | 0⋅88 (0⋅03) | 0⋅86 (0⋅02) | 0⋅91 | 0⋅81 |
| **NPV** | 0⋅48 (0⋅05) | 0⋅48 (0⋅05) | 0⋅27 | 0⋅50 |

**Wellness Scale Models**

Four additional models were constructed using the wellness scale (WS) variable in place of the detailed clinical signs and symptoms: wellness parsimonious (including CT, patient age, body temperature, WS, TTP and CT x TTP), wellness parsimonious without temperature, wellness clinical-only (including patient age, body temperature, and WS), and wellness minimal (including CT, patient age, and WS). The performance indices of these models, obtained from internal validation on the Sierra Leonean patients from the IMC dataset, are shown in Table 5.

All the performance indices of these new models are higher than the corresponding original models. In fact, the performance of the worst performing model in this set, the wellness clinical-only, is comparable to that of the parsimonious model including the detailed signs and symptoms. The calibration curves for the parsimonious models with and without wellness scale are almost indistinguishable, but it is possible to note an improvement for the clinical-only and minimal models with the addition of the wellness scale (Figure 3). To evaluate the effect of imputation of wellness score, we fitted the four wellness models only on those patients with known WS (Suppl. Table S3) and found imputed variance inflation to be minimal.

**Table 5. Validation of the wellness scale models.** These models were evaluated using the same indices of performance as the previous models: AUC, R^2^, Brier, accuracy, sensitivity, and specificity. The means and 95% confidence intervals were obtained with 200 iterations of bootstrap resampling.

|  | **Wellness parsimonious**  **(95% CI)** | **Wellness parsimonious**  **w/out temp. (95% CI)** | **Wellness clinical-only**  **(95% CI)** | **Wellness minimal**  **(95% CI)** |
| --- | --- | --- | --- | --- |
| **AUC** | 0⋅80  (0⋅74, 0⋅84) | 0⋅81  (0⋅76, 0⋅85) | 0⋅74  (0⋅68, 0⋅80) | 0⋅80  (0⋅74, 0⋅84) |
| **R^2^** | 0⋅31  (0⋅24, 0⋅38) | 0⋅30  (0⋅23, 0⋅37) | 0⋅19  (0⋅14, 0⋅25) | 0⋅25  (0⋅19, 0⋅32) |
| **Brier** | 0⋅19  (0⋅13, 0⋅24) | 0⋅18  (0⋅13, 0⋅23) | 0⋅20  (0⋅15, 0⋅26) | 0⋅19  (0⋅13, 0⋅24) |
| **Accuracy** | 0⋅73  (0⋅66, 0⋅79) | 0⋅73  (0⋅67, 0⋅79) | 0⋅69  (0⋅62, 0⋅75) | 0⋅72  (0⋅65, 0⋅77) |
| **Sensitivity** | 0⋅82  (0⋅76, 0⋅86) | 0⋅82  (0⋅77, 0⋅86) | 0⋅79  (0⋅72, 0⋅83) | 0⋅81  (0⋅75, 0⋅85) |
| **Specificity** | 0⋅63  (0⋅55, 0⋅70) | 0⋅63  (0⋅55, 0⋅70) | 0⋅57  (0⋅48, 0⋅64) | 0⋅61  (0⋅53, 0⋅68) |

**
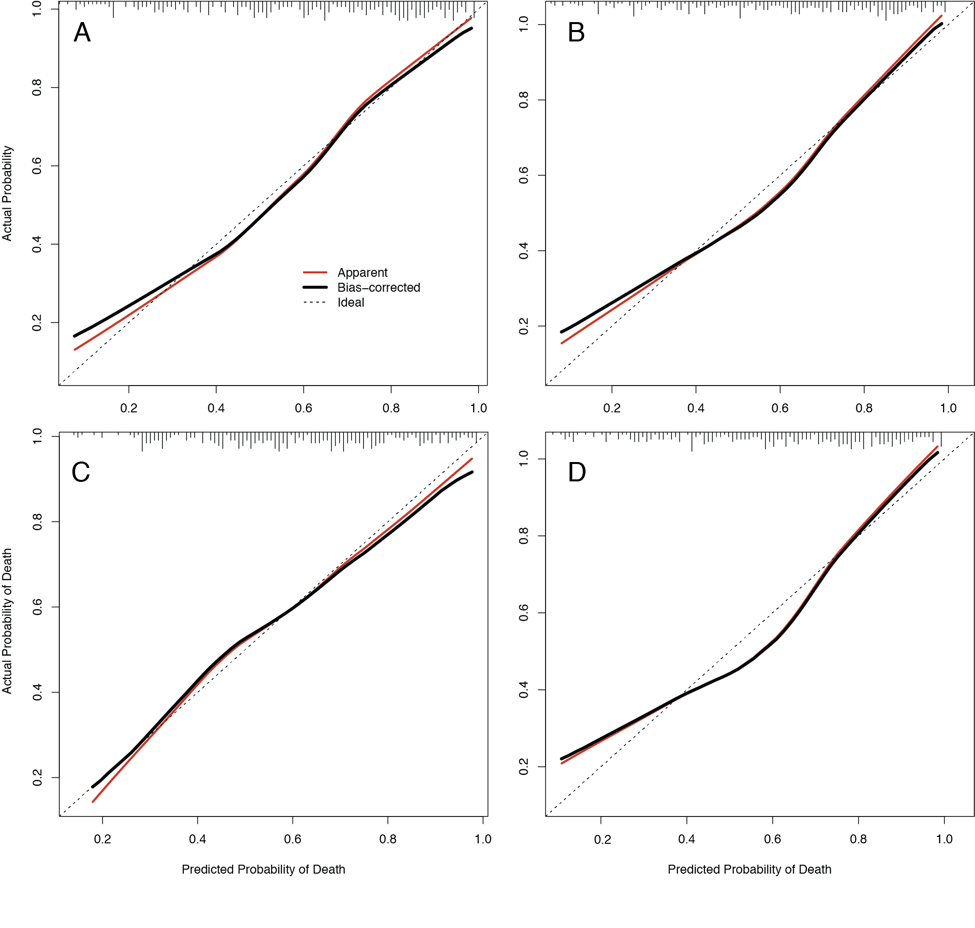
**

**Figure 3. Bootstrap overfitting-corrected calibration curves for the wellness models**. Estimated for the four prognostic models using the wellness scale as predictor: wellness parsimonious (A), wellness parsimonious without body temperature (B), wellness clinical-only (C), and wellness minimal (D). Each plot contains the rug chart at the top showing the distribution of predicted risks. The dotted line represents the apparent calibration curve and the solid line shows the optimism-corrected calibration. A perfectly-calibrated model will fall along the diagonal. Generated with the calibrate function in the rms package.

**Ebola Care Guidelines App**

We created a multilingual (English and French) Android app for health workers, named *Ebola Care Guidelines*, that integrates available patient care and management guidelines for EVD patients with the prognostic models described earlier. To adapt to the needs of rural or remote locations with limited internet access, the app only requires internet connectivity to be installed the first time, and all subsequent use can be performed offline. The home screen shows a list of supportive care recommendations for Ebola patients, compiled from Lamontagne et al. (26), and care and management guidelines for hemorrhagic fevers from WHO (27) and MSF (28) (Figure 4A). The list is categorized by intervention type and selecting a guideline from this list provides a summary description and specific interventions related to that guideline (Figure 4B). When users select a specific intervention, the app redirects to the corresponding page in the reference document (Figure 4C).

Users can also input patient information (age, sex, pregnancy status, weight, height), clinical signs and symptoms recorded at triage, laboratory results, and the wellness scale (WS) from the first clinical rounds after admission (Figure 4D), through a built-in form, or via a separate CommCare (https://dimagi.com/commcare/) app. After all, or some, of this data is recorded, the app computes the severity score of the patient by selecting the model most appropriate for the available data. It then offers a visualization of the score and the magnitude of patient-specific contributions for each feature included in their score, where the score value is shown at the top in a color-graded scale and patient-specific feature contributions to that score are depicted in a bar chart summary page (Figure 4E). Each clinical feature is linked to one or more of the care guidelines according to their appearance in the guideline, so that when that feature is present in the data, the corresponding guidelines are highlighted (Figure 4F). The total severity score can also be linked to specific guidelines when it is over a threshold defined in the app’s settings. Those guidelines will also be highlighted when the score is higher than a user-defined threshold. This feature is designed to bring the user’s attention to the resource-consuming recommendations that could be most relevant given the clinical manifestation of the patient (e.g. intensive monitoring). It is important to note that the app does not make any choices for the user nor does it deviate or omit information from the guidelines, it simply directs the user to the section in the guidelines that advises them in that decision. The trigger signs/symptoms linking to the guidelines are straightforward, since are precisely those listed in the guidelines themselves (e.g. vomiting and diarrhea for the ORS/IV fluids). The app’s source code is available on GitHub under the MIT license. An online wiki provides detailed instructions on how to update the models and the guidelines included in the app (https://github.com/broadinstitute/ebola-imc-public/wiki).

**
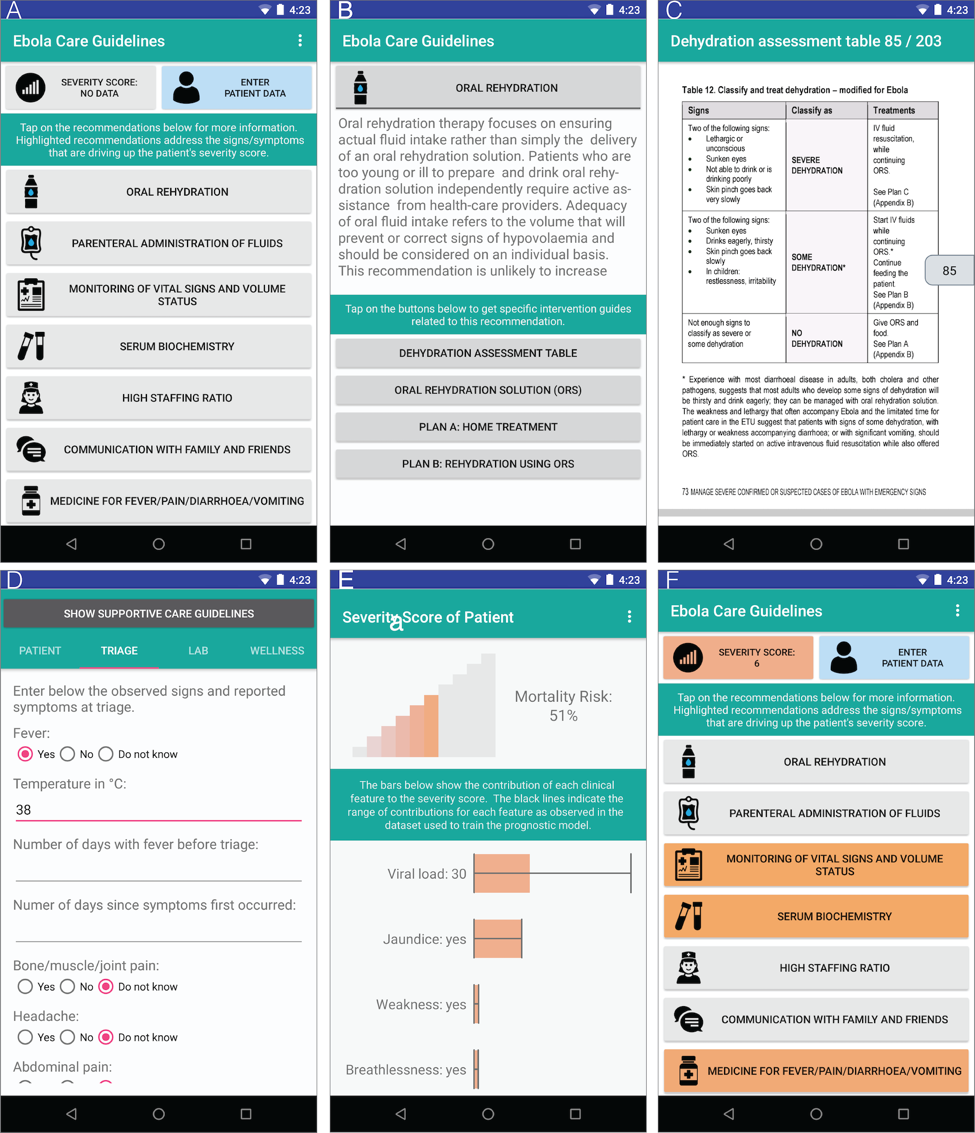
**

**Figure 4. Ebola Care Guidelines app**. The home screen presents the list of recommendations (A), which can be selected to access specific interventions associated to each recommendation (B). Selecting a specific intervention or guideline redirects the user to the corresponding section in the WHO’s manuals for care and management of hemorrhagic fever patients (C). The app allows the users to enter basic demographic information (age, weight), vitals, signs & symptoms at presentation, lab data (CT value from first RT-PCR and malaria test result), and wellness scale (D). Based on the available data, the app calculates the severity score of the patient using the suitable prognostic model and presents a customized risk visualization (E). The recommendations that are associated with the presentation signs and symptoms are highlighted in the home screen (E).

**Discussion**

The purpose of this study was two-fold: first, to present generalizable machine learning prognostic models for EVD derived from the largest multi-center clinical dataset available to date, externally validated across diverse sites representing various periods of the largest Ebola epidemic on record; and second, to show how these models could guide clinical decisions by organizing existing knowledge of patient care and management more efficiently and making it easily available as a mobile app.

**Prognostic Models Recapitulate Prior Findings and Highlight the Importance of Clinical Intuition**

In order to account for different levels of clinical detail collected at the ETUs, we constructed a family of prognostic models that range from models requiring only clinical signs/symptoms or age and viral load, to more complex models incorporating a mixture of laboratory data, signs/symptoms, and observational assessments from experienced health providers. The discriminative capacity of the models is robust across the training set and two independent validation sets, with AUCs ranging from 0⋅75 up to 0⋅80. Furthermore, these models recapitulate several findings reported earlier in the literature and also reveal further associations between mortality and clinical signs/symptoms. The most informative predictors of EVD outcome are patient age and viral load, and models incorporating just these two variables exhibit good performance. Occurrence of jaundice or bleeding at initial presentation are important predictors of death, but both have low incidence at triage among the patients in the IMC cohort of only 5%. In contrast, more widespread EVD manifestations such as dyspnea, dysphagia, and asthenia at triage have a much weaker correlation with mortality. Even though fever is a non-specific symptom with no predictive power, body temperature at triage is informative by increasing specificity in the predictions. The performance of models incorporating the clinical wellness score is at least comparable or superior to the more detailed models including individual clinical features deemed as the most predictive in our variable selection process. This is a particularly interesting result in our study, since it suggests that machine learning approaches, when properly designed and implemented, and applied on rich-enough data, could approximate the clinical intuition that physicians acquire through their experience in the field. Conversely, this also provides evidence that good bedside intuition can very effectively integrate the individual indicators of the patient’s clinical status into an overall health assessment of great predictive power. These models may be useful in emergency situations when the appropriate experience is unavailable or under-developed.

**Updating the Models and App**

Our prognostic models and guidelines app can be easily updated with new data. With increasing knowledge on the management of Ebola, we expect the mortality rate to decrease over time or to be more reliably estimated by earlier symptoms. New information from the ongoing outbreak in DRC or future epidemics would enable us to train and update the models to more appropriate calculations that are adapted to improving standards of care, and geographically divergent variables. For example: health-care seeking behavior in the conflict-afflicted DRC would be undoubtedly more limited than that in West Africa in 2014-16, and perhaps other factors will be more predictive of death. Therefore, not only are the models adapted to the newly available information but the new models would also automatically link up to the easily-updatable guidelines as they become available.

**Data-harmonization Challenges**

When aggregating data from several sources, care must be taken to search and correct for systematic differences between datasets and best ensure interoperability. For example, in order to aggregate CT values and control for analytical bias generated by the different PCR labs, we applied an intra-site normalization to ensure that values were comparable across sites. We had to discard several potentially informative clinical variables such as confusion and coma since they were not available in all the IMC ETUs. Other features could be imputed, such as body temperature by using fever as a proxy variable.

**Validation Strategies**

Our prognostic models, particularly those incorporating the parsimonious sets of predictors, perform well on two independent datasets used for external validation. These datasets have a wide temporal, geographic and clinical scope. A major difference between these datasets was the time during which they were collected, with the KGH data representing an earlier time point, with less refined treatment protocols, increased patient volume and admission intensity with a larger number of patients delayed during transfers from holding centers. On the other hand, the GOAL dataset includes patients from the final months of the epidemic with a 13% lower CFR. Thus, as may be expected, the models underestimated the observed risk for patients of the KGH cohort, while observed risk was slightly overestimated in the GOAL cohort. The IMC training dataset covers a much broader temporal window of the epidemic as well as a wider catchment area, spanning several districts across two countries, which may explain its consistent performance in these disparate populations. We also investigated the temporal flexibility of the models by testing them on CT values obtained two days after triage and comparing model performance on patients with short or long times to presentation at the ETU. The results show that the parsimonious models could maintain their performance parameters when deployed in contexts with systematically different referral times or laboratory turnaround times, and further justifies the use of time-to-presentation within the model to control for time-dependent effects.

**mHealth Applications**

Finally, with the *Ebola Care Guidelines* app we benchmark a robust and flexible mHealth platform for clinicians that could be extended to other diseases affecting rural and low-resource areas. By linking evidence-based prognostication with trusted clinical care information, we propose the app both as a reference tool to improve training and adherence to protocol, as well as a support system that better tailors the prioritization of clinical interventions to the patient's data. The integration of mHealth platforms with rapid point of care diagnostic kits (29, 30) has the potential to realize the concept of a “pocket lab” (31), which could be used outside laboratory settings and during health emergencies. The ultimate goal of these platforms is to aid clinical management decisions by personalizing the understanding of individual prognostic predictions and better organizing access to the trusted, updateable medical knowledge. This platform also allows updating of the prognostic models, where new weightings may be trained on more geographically or temporally relevant data as it becomes available. The current update process simply involves bundling the specifications of the new models (predictor variables and coefficients), rebuilding the app, and releasing it through Android’s app store. Future versions of the app may not even require to be updated, as they could retrieve new models directly from a central server. We believe that if clinical staff can obtain actionable information from the data they collect, they may be incentivized to generate more and higher-quality data: creating a positive feedback loop which drives precision. Further, the visualization of the clinical basis of prediction models (such as the charts provided in our app) provides a learning platform that builds informed clinical experience rather than simply replacing it.

**Limitations**

Despite being the largest EVD prognosis modeling study to date, the amount and quality of available clinical data is still limited. We accounted for these limitations by harmonizing the data from different ETUs and applying various statistical techniques recommended for prognosis modeling (multiple imputation, bootstrap sampling, external validation). Even with the help of these approaches, data might be affected by variations in clinical assessments from clinicians with varying levels of experience, errors in data collection (including patient symptom recall or history taking skills), and differences in lab protocols. Ultimately, future predictive models will require larger and better datasets, and consensus mechanisms to ensure that data is consistent across sites.

**Conclusions**

It is possible to generate generalizable machine learning prognostic models if data from representative cohorts can be harmonized and externally validated. The use of low-cost mHealth tools on the ground incorporating the insights gained from such models, in combination with effective data collection and sharing among all stakeholders, will be key elements in the early detection and containment of future outbreaks of Ebola and other emerging infectious diseases.

**Availability of source code, data, and app**

The full source code is openly available at https://github.com/broadinstitute/ebola-imc-public. Refer to IMC's Ebola Response page (https://internationalmedicalcorps.org/ebola-response), for instructions on how external researchers can access the data. The app is freely available on Google Play: <https://play.google.com/store/apps/details?id=org.broadinstitute.ebola_care_guidelines>.

**Acknowledgments**

We would like to thank the governments of Liberia, Sierra Leone, and Guinea for contributing to the International Medical Corps’ humanitarian response. We would also like to thank all of our generous institutional, corporate, foundation, and individual donors who placed their confidence and trust in International Medical Corps and made our work during the Ebola epidemic possible. We would also like to thank the United States Naval Medical Research Center, Public Health England, the European Union Mobile Laboratory, and the Nigerian Laboratory for providing laboratory data to our Ebola Treatment Units. We would like to further acknowledge all members of our Research Review Committee and other technical teams that contributed to this research. Finally, we would also like to thank our clinical, WASH, and psychosocial teams as well as all of our monitoring and evaluation staff, including the data collection officers at each of our ETUs. AC would like to thank Mary Lynn Baniecki and Christian Matranga for insightful discussions on the EBOV qPCR assays, and Christopher Moxon for critical feedback on the manuscript. Finally, we thank the patients included in this study whose data has and will continue to make invaluable contribution to improving future Ebola care.

**Contributors**

Andres Colubri: project conception, literature search, data analysis, data interpretation, writing, figures, tool development

Mary-Anne Hartley: literature search, data collection, data interpretation, writing, figures

Mathew Siakor: data collection, editing

Vanessa Wolfman: data collection, editing

August Felix: protocol compliance, editing

Tom Sesay: data collection

Jeffrey G. Shaffer: data collection

Robert F. Garry: data collection

Donald S. Grant: data collection

Adam C. Levine: project supervision, data collection, data interpretation, writing

Pardis C. Sabeti: project supervision, data interpretation, writing

**References**

1. Barry A, Ahuka-Mundeke S, Ali Ahmed Y, Allarangar Y, Anoko J, Archer BN, et al. Outbreak of Ebola virus disease in the Democratic Republic of the Congo, April–May, 2018: an epidemiological study. The Lancet. 2018;392(10143):213-21.

2. Moran B. Fighting Ebola in conflict in the DR Congo. The Lancet. 2018;392(10155):1295-6.

3. Fischer WA, Crozier I, Bausch DG, Muyembe J-J, Mulangu S, Diaz JV, et al. Shifting the Paradigm — Applying Universal Standards of Care to Ebola Virus Disease. New England Journal of Medicine. 2019;380(15):1389-91.

4. Amarasingham R, Patel PC, Toto K, Nelson LL, Swanson TS, Moore BJ, et al. Allocating scarce resources in real-time to reduce heart failure readmissions: a prospective, controlled study. BMJ Quality & Safety. 2013;22(12):998-1005.

5. Mitchell M, Hedt-Gauthier BL, Msellemu D, Nkaka M, Lesh N. Using electronic technology to improve clinical care – results from a before-after cluster trial to evaluate assessment and classification of sick children according to Integrated Management of Childhood Illness (IMCI) protocol in Tanzania. BMC Medical Informatics and Decision Making. 2013;13(1):95.

6. Lund S, Boas I, Bedesa T, Fekede W, Nielsen H, Sørensen B. Association between the safe delivery app and quality of care and perinatal survival in ethiopia: A randomized clinical trial. JAMA Pediatrics. 2016;170(8):765-71.

7. Colubri A, Silver T, Fradet T, Retzepi K, Fry B, Sabeti P. Transforming Clinical Data into Actionable Prognosis Models: Machine-Learning Framework and Field-Deployable App to Predict Outcome of Ebola Patients. PLoS neglected tropical diseases. 2016;10(3):e0004549.

8. Schieffelin JS, Shaffer JG, Goba A, Gbakie M, Gire SK, Colubri A, et al. Clinical illness and outcomes in patients with Ebola in Sierra Leone. N Engl J Med. 2014;371(22):2092-100.

9. Roshania R, Mallow M, Dunbar N, Mansary D, Shetty P, Lyon T, et al. Successful Implementation of a Multicountry Clinical Surveillance and Data Collection System for Ebola Virus Disease in West Africa: Findings and Lessons Learned. Global Health: Science and Practice. 2016;4(3):394-409.

10. Hartley M-A, Young A, Tran A-M, Okoni-Williams HH, Suma M, Mancuso B, et al. Predicting Ebola Severity: A Clinical Prioritization Score for Ebola Virus Disease. PLoS neglected tropical diseases. 2017;11(2):e0005265.

11. Hartley M-A, Young A, Tran A-M, Okoni-Williams HH, Suma M, Mancuso B, et al. Predicting Ebola infection: A malaria-sensitive triage score for Ebola virus disease. PLoS neglected tropical diseases. 2017;11(2):e0005356.

12. Gire SK, Goba A, Andersen KG, Sealfon RSG, Park DJ, Kanneh L, et al. Genomic surveillance elucidates Ebola virus origin and transmission during the 2014 outbreak. Science (New York, NY). 2014;345(6202):1369-72.

13. Center USNMR. Ebola Zaire (EZ1) rRT-PCR (TaqMan) Assay. 2014.

14. Trombley AR, Wachter L, Garrison J, Buckley-Beason VA, Jahrling J, Hensley LE, et al. Comprehensive Panel of Real-Time TaqMan™ Polymerase Chain Reaction Assays for Detection and Absolute Quantification of Filoviruses, Arenaviruses, and New World Hantaviruses. The American journal of tropical medicine and hygiene. 2010;82(5):954-60.

15. Prevention CfDCa. Ebola Virus VP40 Real-Time RT-PCR Assay. 2014.

16. Janssen KJM, Donders ART, Harrell FE, Vergouwe Y, Chen Q, Grobbee DE, et al. Missing covariate data in medical research: To impute is better than to ignore. Journal of Clinical Epidemiology. 2010;63(7):721-7.

17. Beaujean A. BaylorEdPsych: R Package for Baylor University Educational Psychology Quantitative Courses. R package version 0.5. 2012.

18. Harrell FE. Hmisc: Harrell Miscellaneous. R package version 4.2. 2019.

19. Harrell FE. Regression modeling strategies : with applications to linear models, logistic and ordinal regression, and survival analysis. Second edition. ed. Cham: Springer; 2015. 1 online resource (xxv, 582 pages) p.

20. Friedman J, Hastie T, Tibshirani R, Simon N, Narasimhan B, Qian J. glmnet: Lasso and Elastic-Net Regularized Generalized Linear Models. R package version 2.0. 2018.

21. Harrell FE. rms: Regression Modeling Strategies. 2019.

22. Saar-Tsechansky M, Provost F. Handling Missing Values when Applying Classification Models. J Mach Learn Res. 2007;8:1623-57.

23. Harel O. The estimation of R 2 and adjusted R 2 in incomplete data sets using multiple imputation. Journal of Applied Statistics. 2009;36(10):1109-18.

24. Zhang J, Yu KF. What's the Relative Risk?A Method of Correcting the Odds Ratio in Cohort Studies of Common Outcomes. JAMA. 1998;280(19):1690-1.

25. Crowe SJ, Maenner MJ, Kuah S, Erickson BR, Coffee M, Knust B, et al. Prognostic Indicators for Ebola Patient Survival. Emerging Infectious Disease journal. 2016;22(2):217.

26. Lamontagne F, Fowler RA, Adhikari NK, Murthy S, Brett-Major DM, Jacobs M, et al. Evidence-based guidelines for supportive care of patients with Ebola virus disease. The Lancet. 2018;391(10121):700-8.

27. WHO. Manual for the care and management of patients in Ebola care units / Community Care Centres: interim emergency guidance, January 2015. 2015.

28. WHO. Clinical management of patients with viral haemorrhagic fever. A pocket guide for front-line health workers. 2016.

29. Broadhurst MJ, Kelly JD, Miller A, Semper A, Bailey D, Groppelli E, et al. ReEBOV Antigen Rapid Test kit for point-of-care and laboratory-based testing for Ebola virus disease: a field validation study. The Lancet.386(9996):867-74.

30. Yen C-W, de Puig H, Tam JO, Gomez-Marquez J, Bosch I, Hamad-Schifferli K, et al. Multicolored silver nanoparticles for multiplexed disease diagnostics: distinguishing dengue, yellow fever, and Ebola viruses. Lab on a Chip. 2015;15(7):1638-41.

31. Radin JM, Topol EJ, Andersen KG, Steinhubl SR. A laboratory in your pocket. The Lancet.388(10054):1875.

**Machine-Learning prognostic models from the 2014-16 Ebola outbreak: data-harmonization challenges, validation strategies, and mHealth applications**

Andres Colubri, Mary-Anne Hartley, Mathew Siakor, Vanessa Wolfman, August Felix, Tom Sesay, Jeffrey G. Shaffer, Robert F. Garry, Donald S. Grant, Adam C. Levine, Pardis C. Sabeti

**Supplementary materials**

**
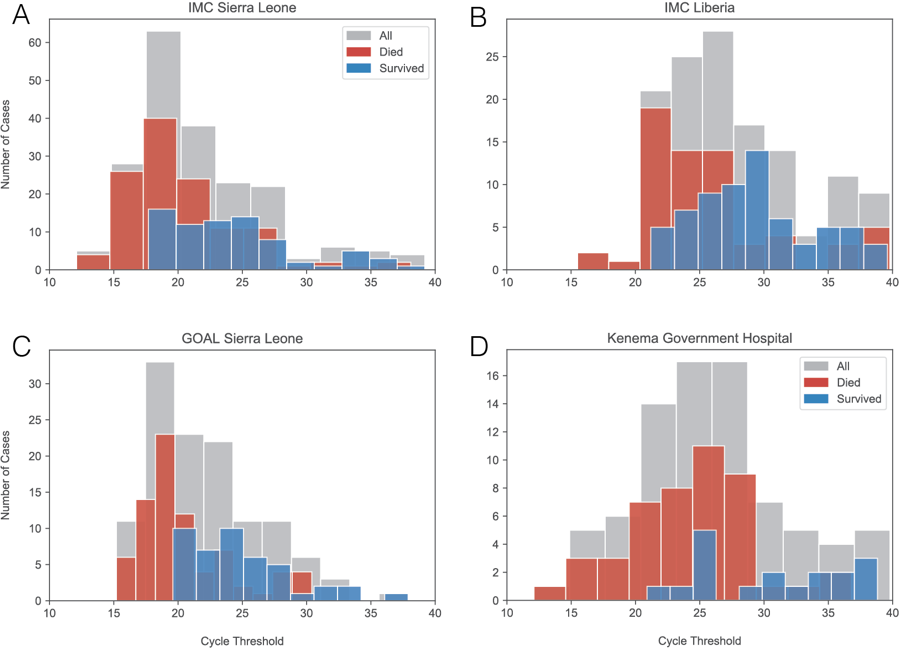
**

**Figure S1: Distribution of RT-PCR Cycle Threshold (CT) across all sites.** The histograms represent the distributions of the CT values from all the sites considered for model training and validation:(A) ETUs operated by IMC in Sierra Leone, located at Lunsar (Port Loko District), Kambia (Kambia District), and Makeni (Bombali District). (B) ETUs operated by IMC Liberia, located at Suakoko (Bong County) and Kakata (Margibi County). (C) GOAL-Mathaska ETU in Port Loko, Sierra Leone. (D) Kenema Government Hospital in Sierra Leone. In each plot, the grey, red and blue histograms represent all, fatal and surviving patients respectively.


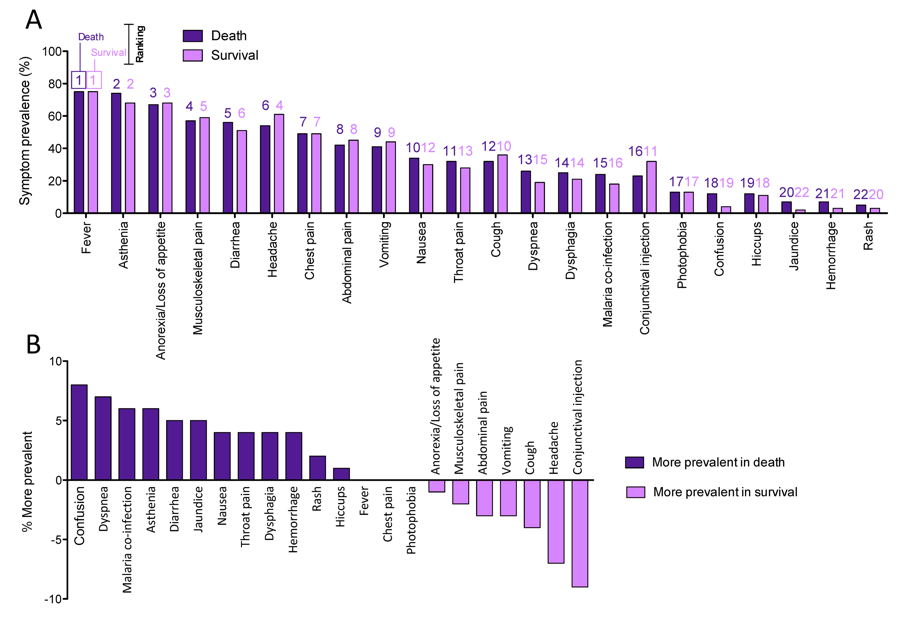


**Figure S2: Prevalence of clinical signs and symptoms recorded at triage***.* (A) Prevalence of clinical characteristics at triage amongst Ebola patients who either survived or died, ranked according to the prevalence in fatal outcomes. Rankings from 1–22 are listed above each bar: purple for the outcome of death and pink for survival. (B) Differences in symptom prevalence between EVD survivors and those who died. Positive values are more prevalent in fatal outcomes. Negative values are more prevalent in survivors.

**
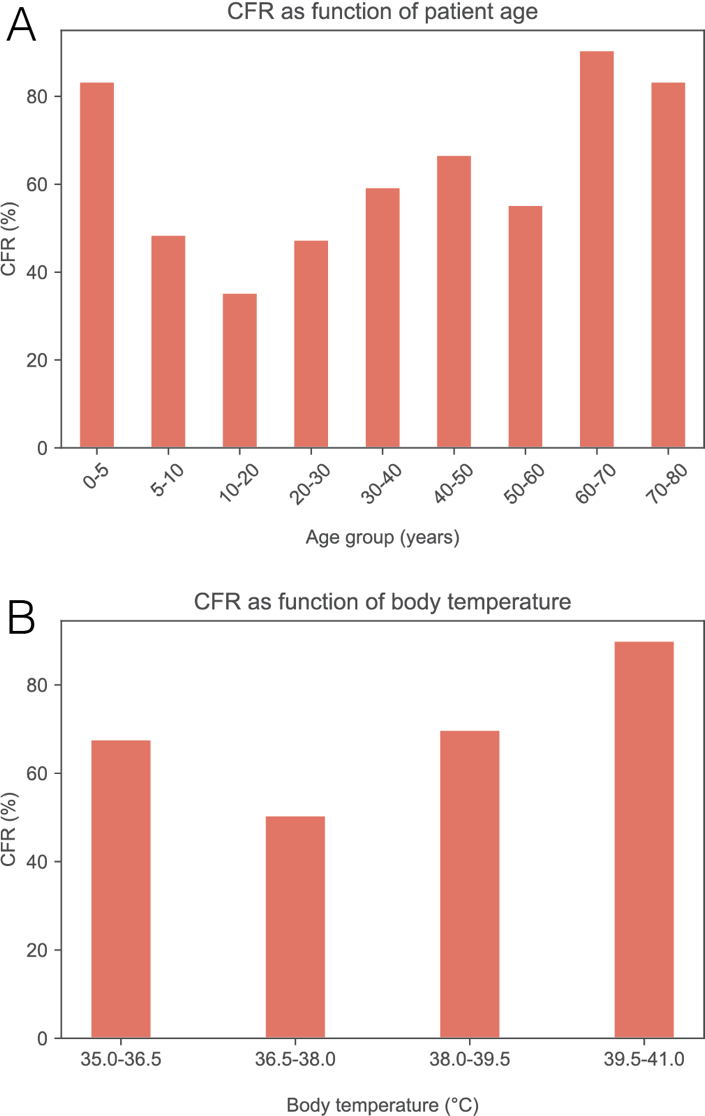
**

**Figure S3: Case fatality rate as a function of patient age and body temperature.** Case Fatality Rate (CFR) as a function of (A) patient age and (B) body temperature at triage. Age was binned in 5-year intervals, and temperature in 1.5 °C intervals.

**
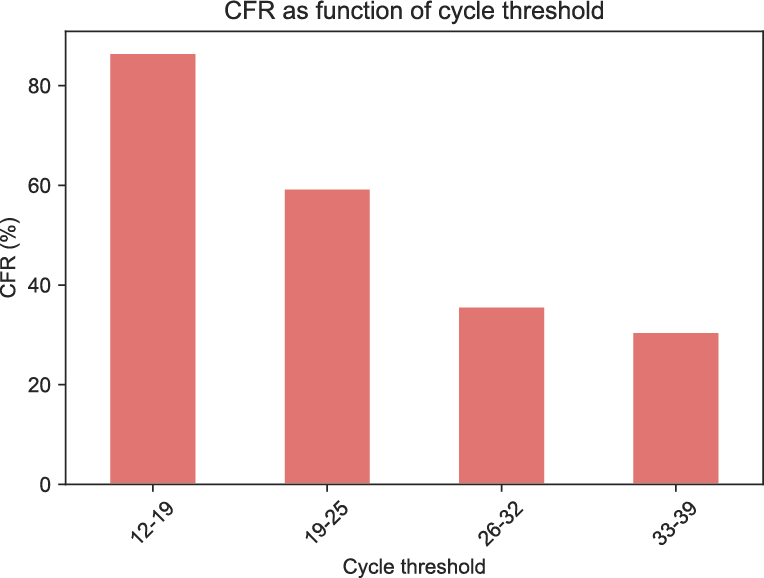
**

**Figure S4: Case fatality rate as a function of cycle threshold***.* The CT values were grouped in 5 bins between the minimum and maximum observed values.

**Table S1: Model specifications.** Tables describing all the models constructed in this paper: (A) parsimonious, (B) parsimonious without body temperature, (C) clinical-only, (D) minimal, (E) wellness scale parsimonious, (F) wellness scale parsimonious without body temperature, (G) wellness scale clinical-only, and (H) wellness scale minimal. The tables provide the model coefficients for each term, their P-values, odds ratios (OR) for the linear terms (caused by the presence of a sign/symptom or an IQR change in continuous variables), risk ratios (RR, estimated from the odds ratios only for categorical variables), type of term (constant, linear, restricted cubic spline or RCS, and product), and the knots of the non-linear RCS term (RCS1). The formula of the logit(p) function for each model is provided as well.

CT : cycle threshold value of Ebola-specific RT-PCR, TTP: Time to presentation

**S1A: Parsimonious model**

| **Term** | **Coefficient** | **P-value** | **OR** | **RR** | **Type** | **Knots** |
| --- | --- | --- | --- | --- | --- | --- |
| **Intercept** | 8.14656178 | 0.5795 |  |  | constant |  |
| **Patient Age** | -0.0588981 | 0.0004 |  |  | RCS0 |  |
| **Patient Age'** | 0.10325771 | <0.0001 |  |  | RCS1 | 5 30 58 |
| **CT** | -0.6919651 | 0.0013 | 0.42 |  | linear |  |
| **Body Temperature** | -0.2056551 | 0.6050 |  |  | RCS0 |  |
| **Body Temperature'** | 0.75788984 | 0.2541 |  |  | RCS1 | 36.3 37.2 39.31 |
| **Jaundice** | 1.33911639 | 0.0360 | 3.82 | 1.04 | linear |  |
| **Bleeding** | 1.02004343 | 0.0511 | 2.77 | 1.04 | linear |  |
| **Dyspnea** | 0.17193284 | 0.5388 | 1.19 | 1.03 | linear |  |
| **Dysphagia** | 0.14673348 | 0.5992 | 1.16 | 1.03 | linear |  |
| **TTP** | 0.00119668 | 0.9759 | 1.01 |  | linear |  |
| **CT x TTP** | -0.0420558 | 0.2926 |  |  | product |  |

**Formula:** 8.14656 - 0.0589 age + 4x10^-5^ max(age - 5.0, 0)^3^ – 7x10^-5^ max(age - 30.0, 0)^3^ + 3x10^-5^ max(age - 58.0, 0)^3^ - 0.69197 CT - 0.20566 temp + 0.08365 max(temp - 36.3, 0)^3^ - 0.11933 max(temp - 37.2, 0)^3^ + 0.03568 max(temp - 39.31, 0)^3^ + 1.33912 jaundice + 1.02004 bleeding + 0.17193 dyspnea + 0.14673 dysphagia + 0.0012 TTP - 0.04206 CT x TTP

**S1B: Parsimonious model without body temperature**

| **Term** | **Coefficient** | **P-value** | **OR** | **RR** | **Type** | **Knots** |
| --- | --- | --- | --- | --- | --- | --- |
| **Intercept** | 0.79342334 | 0.0120 |  |  | constant |  |
| **Patient Age** | -0.0596321 | 0.0003 |  |  | RCS0 |  |
| **Patient Age'** | 0.10436838 | <0.0001 |  |  | RCS1 | 5 30 58 |
| **CT** | -0.6944901 | 0.0011 | 0.42 |  | linear |  |
| **Jaundice** | 1.31695855 | 0.0373 | 3.72 | 1.04 | linear |  |
| **Bleeding** | 0.92970184 | 0.0725 | 2.53 | 1.03 | linear |  |
| **Dyspnea** | 0.16771205 | 0.5458 | 1.18 | 1.04 | linear |  |
| **Dysphagia** | 0.08827503 | 0.7442 | 1.09 | 1.02 | linear |  |
| **TTP** | -0.0023547 | 0.9515 | 1.00 |  | linear |  |
| **CT x TTP** | -0.04104 | 0.3011 |  |  | product |  |

**Formula:** 0.79342 - 0.05963 age + 4x10^-5^ max(age - 5.0, 0)^3^ – 7x10^-5^ max(age - 30.0, 0)^3^ + 3e-05 max(age - 58.0, 0)^3^ - 0.69449 CT + 1.31696 jaundice + 0.9297 bleeding + 0.16771 dyspnea + 0.08828 dysphagia - 0.00235 TTP - 0.04104 CT x TTP

**S1C: Clinical-only model**

| **Term** | **Coefficient** | **P-value** | **OR** | **RR** | **Type** | **Knots** |
| --- | --- | --- | --- | --- | --- | --- |
| **Intercept** | 3.02919786 | 0.8213 |  |  | constant |  |
| **Patient Age** | -0.0527675 | 0.0004 |  |  | RCS0 |  |
| **Patient Age'** | 0.08708779 | <0.0001 |  |  | RCS1 | 5 30 58 |
| **Body Temperature** | -0.0752819 | 0.8355 |  |  | RCS0 |  |
| **Body Temperature'** | 0.57465845 | 0.3436 |  |  | RCS1 | 36.3 37.2 39.31 |
| **Jaundice** | 1.30197731 | 0.0241 | 3.68 | 1.03 | linear |  |
| **Bleeding** | 1.16552489 | 0.0149 | 3.21 | 1.03 | linear |  |
| **Dyspnea** | 0.16085186 | 0.5328 | 1.17 | 1.04 | linear |  |
| **Dysphagia** | 0.27387136 | 0.2891 | 1.31 | 1.05 | linear |  |
| **Asthenia/weakness** | 0.23194879 | 0.3249 | 1.26 | 1.16 | linear |  |
| **Diarrhea** | 0.14197065 | 0.5163 | 1.15 | 1.07 | linear |  |

**Formula:** 3.0292 - 0.05277 age + 3x10^-5^ max(age - 5.0, 0)^3^ - 6e-05 max(age - 30.0, 0)^3^ + 3x10^-5^ max(age - 58.0, 0)^3^ - 0.07528 temp + 0.06343 max(temp - 36.3, 0)^3^ - 0.09048 max(temp - 37.2, 0)^3^ + 0.02705 max(temp - 39.31, 0)^3^ + 1.30198 jaundice + 1.16552 bleeding + 0.16085 dyspnea + 0.27387 dysphagia + 0.23195 asthenia + 0.14197 diarrhea

**S1D: Minimal model**

| **Term** | **Coefficient** | **P-value** | **OR** | **RR** | **Type** | **Knots** |
| --- | --- | --- | --- | --- | --- | --- |
| **Intercept** | 0.86215216 | 0.0042 |  |  | constant |  |
| **Patient Age** | -0.0534333 | 0.0007 |  |  | RCS0 |  |
| **Patient Age'** | 0.09478786 | <0.0001 |  |  | RCS1 | 5 30 58 |
| **CT** | -0.8694039 | <0.0001 | 0.34 |  | linear |  |

**Formula:** 0.86215 - 0.05343 age + 3x10^-5^ max(age - 5.0, 0)^3^ – 6x10^-5^ max(age - 30.0, 0)^3^ + 3x10^-5^ max(age - 58.0, 0)^3^ - 0.8694 CT

**S1E: Wellness scale parsimonious model**

| **Term** | **Coefficient** | **P-value** | **OR** | **RR** | **Type** | **Knots** |
| --- | --- | --- | --- | --- | --- | --- |
| **Intercept** | 21.379251 | 0.2201 |  |  | constant |  |
| **Patient Age** | -0.0754931 | 0.0014 |  |  | RCS0 |  |
| **Patient Age'** | 0.13750982 | <0.0001 |  |  | RCS1 | 4 28 60 |
| **CT** | -0.5247369 | 0.0862 | 0.51 |  | linear |  |
| **Body Temperature** | -0.5798065 | 0.2196 |  |  | RCS0 |  |
| **Body Temperature'** | 1.25110525 | 0.1077 |  |  | RCS1 | 36.2 37.2 39.29 |
| **Wellness Scale** | 0.49509775 | 0.0142 | 2.69 |  | linear |  |
| **TPP** | -0.0930255 | 0.1502 | 0.39 |  | linear |  |
| **CT x TTP** | -0.106844 | 0.2122 |  |  | product |  |

**Formula:** 21.37925 - 0.07549 age + 4x10^-5^ max(age - 4.0, 0)^3^ - 8x10^-5^ max(age - 28.0, 0)^3^ + 3x10^-5^ max(age - 60.0, 0)^3^ - 0.52474 CT - 0.57981 temp + 0.13103 max(temp - 36.2, 0)^3^ - 0.19373 max(temp - 37.2, 0)^3^ + 0.06269 max(temp - 39.29, 0)^3^ + 0.4951 WS - 0.09303 TTP - 0.10684 CT x TTP

**S1F: Wellness scale parsimonious without body temperature model**

| **Term** | **Coefficient** | **P-value** | **OR** | **RR** | **Type** | **Knots** |
| --- | --- | --- | --- | --- | --- | --- |
| **Intercept** | 0.17095348 | 0.8039 |  |  | constant |  |
| **Patient Age** | -0.0749107 | 0.0012 |  |  | RCS0 |  |
| **Patient Age'** | 0.1384085 | <0.0001 |  |  | RCS1 | 4 28 60 |
| **Cycle Threshold** | -0.5033504 | 0.0930 | 0.53 |  | linear |  |
| **Wellness Scale** | 0.50788889 | 0.0108 | 2.76 |  | linear |  |
| **Time to Presentation** | -0.0968393 | 0.1256 | 0.38 |  | linear |  |
| **CT x TTP** | -0.1070374 | 0.2070 |  |  | product |  |

**Formula:** 0.17095 - 0.07491 age + 4x10^-5^ max(age - 4.0, 0)^3^ - 8x10^-5^ max(age - 28.0, 0)^3^ + 3x10^-5^ max(age - 60.0, 0)^3^ - 0.50335 CT + 0.50789 WS - 0.09684 TTP - 0.10704 CT x TTP

**S1G: Wellness scale clinical-only model**

| **Term** | **Coefficient** | **P-value** | **OR** | **RR** | **Type** | **Knots** |
| --- | --- | --- | --- | --- | --- | --- |
| **Intercept** | 13.0101569 | 0.3890 |  |  | constant |  |
| **Patient Age** | -0.0691352 | 0.0006 |  |  | RCS0 |  |
| **Patient Age'** | 0.123125 | <0.0001 |  |  | RCS1 | 4 28 60 |
| **Body Temperature** | -0.3840269 | 0.3494 |  |  | RCS0 |  |
| **Body Temperature'** | 0.99239011 | 0.1543 |  |  | RCS1 | 36.2 37.2 39.29 |
| **Wellness Scale** | 0.74786034 | <0.0001 | 2.11 |  | linear |  |

**Formula:** 13.01016 - 0.06914 age + 4x10^-5^ max(age - 4.0, 0)^3^ - 7x10^-5^ max(age - 28.0, 0)^3^ + 3x10^-5^ max(age - 60.0, 0)^3^ - 0.38403 temp + 0.10394 max(temp - 36.2, 0)^3^ - 0.15367 max(temp - 37.2, 0)^3^ + 0.04973 max(temp - 39.29, 0)^3^ + 0.74786 WS

**S1H: Wellness scale minimal model**

| **Term** | **Coefficient** | **P-value** |  |  | **Type** | **Knots** |
| --- | --- | --- | --- | --- | --- | --- |
| **Intercept** | 0.17627324 | 0.7957 |  |  | constant |  |
| **Patient Age** | -0.0826796 | 0.0002 |  |  | RCS0 |  |
| **Patient Age'** | 0.14654649 | <0.0001 |  |  | RCS1 | 4 28 60 |
| **Cycle Threshold** | -0.7917185 | 0.0002 | 0.38 |  | linear |  |
| **Wellness Scale** | 0.43591077 | 0.0198 | 2.39 |  | linear |  |

**Formula:** 0.17627 - 0.08268 age + 5x10^-5^ max(age - 4.0, 0)^3^ - 8x10^-5^ max(age - 28.0, 0)^3^ + 4x10^-5^ max(age - 60.0, 0)^3^ - 0.79172 CT + 0.43591 WS

**Table S2: Temporal flexibility of models.** Performance indices of the parsimonious model when evaluated on patients with day 0 CT values and day 2 CT values (A). Table B shows the performance of the parsimonious model on early comers (TTP <= 3) and late comers (TTP > 3) (B). In this case, CT values were obtained up to 48 hours after admission.

**A**

|  | **Parsimonious**  **with d0 CT** | **Parsimonious**  **with d2 CT** |
| --- | --- | --- |
| **AUC** | 0⋅88 | 0⋅78 |
| **Brier** | 0⋅14 | 0⋅22 |
| **Accuracy** | 0⋅82 | 0⋅79 |
| **Sensitivity** | 0⋅89 | 0⋅78 |
| **Specificity** | 0⋅71 | 0⋅80 |

**B**

|  | **Parsimonious**  **on early-comers (TTP <= 3)** | **Parsimonious**  **on late-comers (TTP > 3days)** |
| --- | --- | --- |
| **AUC** | 0⋅84 | 0⋅92 |
| **Brier** | 0⋅16 | 0⋅13 |
| **Accuracy** | 0⋅83 | 0⋅81 |
| **Sensitivity** | 0⋅85 | 0⋅92 |
| **Specificity** | 0⋅79 | 0⋅62 |

**Table S3: Performance indices of wellness models without imputation.** Performance indices of the wellness scale models obtained without applying multiple imputation on the wellness scale.

|  | **WS parsimonious**  **(95% CI)** | **WS parsimonious**  **w/out temp.**  **(95% CI)** | **WS clinical-only**  **(95% CI)** | **WS minimal**  **(95% CI)** |
| --- | --- | --- | --- | --- |
| **AUC** | 0.80 (0.74, 0.84) | 0.80 (0.75, 0.85) | 0.74 (0.67, 0.79) | 0.80 (0.74, 0.85) |
| **R2** | 0.31 (0.24, 0.38) | 0.30 (0.23, 0.37) | 0.20 (0.15, 0.26) | 0.25 (0.19, 0.32) |
| **Brier** | 0.19 (0.13, 0.24) | 0.18 (0.13, 0.24) | 0.21 (0.15, 0.27) | 0.18 (0.13, 0.24) |
| **Accuracy** | 0.73 (0.66, 0.79) | 0.72 (0.66, 0.78) | 0.68 (0.60, 0.74) | 0.72 (0.65, 0.77) |
| **Sensitivity** | 0.81 (0.75, 0.85) | 0.81 (0.76, 0.86) | 0.78 (0.72, 0.84) | 0.81 (0.75, 0.85) |
| **Specificity** | 0.64 (0.56, 0.71) | 0.61 (0.53, 0.68) | 0.55 (0.47, 0.63) | 0.60 (0.52, 0.67) |

**PCR Lab Notes**

Liberia

For both the Bong and Margibi ETUs, laboratory diagnosis of Ebola virus disease was performed at the United States Naval Medical Research Center (NMRC) Mobile Laboratory in Bong County, Liberia, with the 1-step quantitative Ebola Zaire real-time reverse transcriptase–polymerase chain reaction (TaqMan) assay (Naval Medical Research Center, Frederick, MD). Briefly, Qiagen buffer AVL/ethanol-inactivated blood samples were extracted with QIAamp Viral RNA Mini Kit. Extracted ribonucleic acid was tested for 2 Ebola virus disease gene targets (EBOV Zaire locus and minor groove binding locus), using the Applied Biosystems StepOnePlus instrument. A sample was confirmed to be positive for Ebola virus disease if both targets were detected.

Sierra Leone

The Public Health England (PHE) Port Loko lab (serving Lunsar ETU) switched Ebola real-time PCR assay from commercial Altona –widely used by other “mobile” labs in West Africa – to the more sensitive and robust in-house “Trombley” on 2/3/15. The Public Health England Makeni lab (serving Makeni ETU) had made the switch on 2/4/15.  Both assays tested a single EVD gene target. The Nigerian Lab (serving Kambia ETU) used the Altona assay the entire time.

Briefly, the process used was that the plasma sample (80ul) was inactivated in Qiagen AVL (320 µl) /10mins, then heat-treated for 15 min at 60C, prior to extraction using a Qiagen EZ1 virus kit V2.0. The eluted nucleic acid was analysed using a one-step real time PCR directed against the EBOV Zaire nucleoprotein region (based on the PCR by Trombley et al [3], with some modifications including multiplexing with an Internal control [1, 2]), and performed on a Smartcycler PCR machine.

The NRMC lab assays and processing methodologies are fairly similar to the PHE labs. The NRMC use two targets, including the EBOV Zaire target and the minor groove binding target, whereas the PHE lab used only the EBOV Zaire target. The other main difference was the PHE extraction machine was automated whereas the NRMC was a manual methodology, but both would be expected to generate similar results.

More specific details are in the papers below.

1.      **Weller SA, Bailey D, Matthews S, Lumley S, Sweed A, Ready D, Eltringham G, Richards J, Vipond R, Lukaszewski R, Payne PM, Aarons E, Simpson AJ, Hutley EJ, Brooks T.** 2016. Evaluation of the Biofire FilmArray BioThreat-E Test (v2.5) for Rapid Identification of Ebola Virus Disease in Heat-Treated Blood Samples Obtained in Sierra Leone and the United Kingdom. J Clin Microbiol **54:**114-119.

2.      **Broadhurst MJ, Kelly JD, Miller A, Semper A, Bailey D, Groppelli E, Simpson A, Brooks T, Hula S, Nyoni W, Sankoh AB, Kanu S, Jalloh A, Ton Q, Sarchet N, George P, Perkins MD, Wonderly B, Murray M, Pollock NR.** 2015. ReEBOV Antigen Rapid Test kit for point-of-care and laboratory-based testing for Ebola virus disease: a field validation study. Lancet **386:**867-874.

3.      **Trombley AR, Wachter L, Garrison J, Buckley-Beason VA, Jahrling J, Hensley LE, Schoepp RJ, Norwood DA, Goba A, Fair JN, Kulesh DA.** 2010. Comprehensive panel of real-time TaqMan polymerase chain reaction assays for detection and absolute quantification of filoviruses, arenaviruses, and New World hantaviruses. Am J Trop Med Hyg **82:**954-960.
